# Paediatric emergency medicine practice in Nigeria: a narrative review

**DOI:** 10.1186/s12873-023-00790-1

**Published:** 2023-03-16

**Authors:** Joy N. Eze, Benedict O. Edelu, Ikenna K. Ndu, Tagbo Oguonu

**Affiliations:** 1 Department of Pediatrics, College of Medicine, University of Nigeria/University of Nigeria Teaching Hospital, Ituku Ozalla, Enugu, 400001 Nigeria; 2 University of Nigeria Teaching Hospital, Ituku-Ozalla, Enugu, Nigeria; 3 Department of Paediatrics, College of Medicine, Enugu State University of Science and Technology, Enugu, Nigeria; 4 Enugu State University of Science and Technology Teaching Hospital, Enugu, Nigeria

**Keywords:** Paediatric, Emergency medicine, Practice, Quality of service, Capacity needs, Funding, Nigeria

## Abstract

The practice of paediatric emergency medicine in Nigeria is still evolving, and laden with enormous challenges which contribute to adverse outcomes of childhood illnesses in emergency settings. Deaths from childhood illnesses presenting as emergencies contribute to overall child mortality rates in Nigeria. This narrative review discusses existing structures, organization, and practice of paediatric emergency in Nigeria. It highlights some of the challenges and suggests ways of surmounting them in order to reduce deaths in the children emergency units in Nigerian hospitals. Important aspects of this review include current capacity and need for capacity development, equipment needs for emergency care, quality of service in the context of inadequate healthcare funding and the need for improvement.

## Background

The practice of paediatric emergency in low-middle income countries (LMICs), particularly in sub-Saharan Africa (SSA) has remained daunting owing to a lack of skilled manpower, infrastructure, and equipment [1–3]. Although the special needs of children in SSA are well recognized, adequate response to the needs has not been properly established. This is partly due to inadequacies in the entire health care system attributable to poor healthcare financing, particularly in emergency care. The problem is compounded by substantial gaps in the availability of morbidity and mortality data especially from the rural areas [3, 4]; which has made it difficult to establish the magnitude of the problem and convince policymakers to make major new investments in paediatric emergency care [3]. For these reasons, providing timely, high-quality care for the initial management of critically ill children in African hospitals remains a challenge [1]. The overall quality of care differs between countries and among hospitals. Services are generally better in tertiary facilities than in secondary or primary care facilities because of better working conditions for health workers; and relatively better availability of basic utilities and equipment. Despite the variations in availability and quality of services among the three levels of healthcare, the mortality rates in the children’s emergency room remain high [1, 3]. More than 50% of deaths recorded in children’s emergency room in resource-limited settings occur within the first 24 h of admission [2, 5, 6]. These deaths are mostly from treatable conditions, and occur partly because of late presentation and inadequate hospital service provision on arrival [2].

Childhood morbidity and mortality could be reduced by provision of standardised emergency care for paediatric emergencies. The standards would ensure that necessary human resource, infrastructure, and equipment are clear to all those that are tasked with the provision of paediatric emergent care. The standards can detail the training and re-training of practitioners in the requisite knowledge and skills; infrastructural or facility development, provision of appropriate equipment in adequate numbers; as well as policy prioritization and adequate funding for sustainability by all stakeholders [1, 7, 8]. In addition, audit of the current standards can highlight areas of deficiency, identify potential targets for process improvement and ultimately lead to improved patient outcomes [1]. Studies in Malawi, Kenya, and Tanzania have shown that improving the systems and training in paediatric emergency care can significantly reduce in-hospital mortality [9–11]. Recent studies have shown that Nigeria’s health service is far from being optimally designed and prepared to deliver optimal emergency care to its children [12, 13]. Paediatric emergency units of most hospitals in Nigeria are often understaffed and ill-equipped and are run by doctors and nurses who have no formalised training in paediatric emergency care. The situation is worse for hospitals situated in rural areas and privately owned hospitals. We aimed to review the existing structures, organization, and practice, challenges and prospects of paediatric emergency medicine practice in Nigeria; and proffer possible ways of improving this practice in order to bridge the gaps in providing the initial holistic care of children under emergency conditions.

## Global perspectives of paediatric emergency medicine practice: an overview

Despite the gap in training capacity between the LMICs and high- income countries, maintaining a baseline standard of care remains an overarching problem not just in LMIC but also in high-income countries [14–16]. Emergency preparedness of hospitals in most of these countries is deemed suboptimal. In the United States of America, an ED that maintains a baseline level of pediatric resources in keeping with the national guidelines developed by the American Academy of Pediatrics in collaboration with the American College of Emergency Physicians is considered pediatric-ready[16]. Despite the efforts and resources devoted to training and monitoring in paediatric emergency medicine practice in the United States, compliance with the paediatric emergency medicine guidelines is not optimal[17, 18]. In England, UK, 28% of acute hospital trusts were “weak” for children’s emergency services[19].

Compared to high-income countries, paediatric emergency care is among the weakest parts of health systems in low-income countries in both quality and accessibility [12, 14]. Very few facilities in LMIC have dedicated PEDs. Obermeyer and colleagues observed that only 36 (19%) of 192 emergency medicine facilities in LMICs were designated for children [3]. Hospitals in LMICs are significantly poor compared to those in the developed world given problems such as lower staffing ratios, lack of skills and resources, lack of essential equipment for emergency care and higher acuity of patients [12, 14, 16, 20, 21]. The majority of emergency care in most African countries is still provided by physicians and mid-level practitioners with no formal EM training and little pediatric-specific training [9]. The problem is compounded by lack of basic equipment. Overall, the median availability of functional equipment for resuscitation in emergency settings remains below 50% in some African hospitals [12, 20]. In order to improve the quality of emergency medicine practice, concerted efforts need to be made to maintain a baseline standard of care in emergency settings, particularly in LMICs including Nigeria. This can only be achieved if stakeholders understood the magnitude of the problem.

## Current state of paediatric emergency medicine structure and practice in Nigeria: an overview

### Infrastructure and organisation

The Nigerian healthcare system is organised into primary, secondary and tertiary healthcare levels. Of the 40,348 operational health facilities in Nigeria; 85.1% are primary, 14.5% secondary and 0.4% tertiary [22]. Secondary- and tertiary healthcare facilities are mostly found in urban areas, whereas rural areas are predominantly served by primary health care (PHC) facilities [23]. The imbalance in the structural and geographical distribution of the hospitals and health centers between the urban and rural areas give rise to inequitable provision of health services including emergency services. Given the inequality in facility distribution and service delivery, paediatric emergency medicine practice in the primary and secondary care centers is grossly suboptimal. This has brought undue pressures on the tertiary care centers which are also not optimally ready for quality service. Structural facilities and equipment in the children’s emergency wards in almost all the tertiary centres in Nigeria are grossly inadequate [12, 13]. These range from emergency rooms not well structured for easy access and workflow, absence of triage and resuscitation areas, side laboratory, dedicated pharmacy and radiological facility, to absence or at best broken-down equipment. Due to limited spaces, emergency treatment areas in hospitals are often crowded and hamper patient flow in and out of the emergency room. The triage areas where present are usually not spacious enough. Studies have noted that hospitals in less developed countries lacked an adequate system for triage; and most emergency treatment areas were poorly organized [2, 21, 24]. Initial patient assessment in this circumstance was often inadequate and treatments are delayed. Molyneux made a very pertinent suggestion for planners to consider the way patients are received and moved through the department to obtain different aspects of care and the best way to improve timely patient care and supervision without causing bottlenecks or confusion [2]. Ideally, patients should enter through one doorway and exit through another, with services arranged in sequence of use to avoid counter flows of patients across the corridors [2].

The problem of space in paediatric emergency rooms of hospitals in Nigeria is compounded by the absence of high dependency care areas for managing critically ill children. Dedicated paediatric intensive care units are absent in most hospitals in Nigeria. Thus children who need intensive care admission would remain in the emergency room clogging the already limited space. A pioneer Paediatric Intensive Care Unit (PICU) project was recently initiated at the University of Nigeria Teaching Hospital, Ituku-Ozalla Enugu, south-east, Nigeria by alumnus of the University of Nigeria, College of Medicine [25]. Similar PICU project has also been established at the University College Hospital Ibadan. These are laudable projects enabling enhanced care of children who need ICU admission. It is hoped that other hospitals in Nigeria will emulate these great initiatives.

### Human resource and quality of service

Capacity development of all cadres of staff across all levels of health care is a requisite for delivery of quality health service. Large disparities in the distribution of the health workforce and skills exist between rural and urban areas in Nigeria. Poor attraction and retention of health workers in the rural areas have resulted in inequitable distribution of health workers and access to quality health services at the primary health centers [23]. The PHCs are run by staffs that lack the necessary skills to resuscitate a child under emergency settings. Although the secondary and tertiary care hospitals are better staffed, lack of basic skills needed for efficient emergency service delivery are very apparent among healthcare professionals in these hospitals. Only 55.6% of the doctors and none of the nurses in the study by Paul and Edelu had the requisite certification in basic emergency skills [13]. The situation has remained the same given the recent reports by Enyuma et al., of an overall deficiency in emergency care preparedness amongst PEDs in tertiary care facilities in Nigeria. The authors observed that none of the paediatricians heading the PEDs had a subspecialist/fellowship qualification in emergency medicine; and only 11.8% of the nurses had any certification in emergency care skills [12]. It then buttresses the fact that Nigeria needs to take skill acquisition training for all health care providers seriously, especially those that work in emergency settings.

Paediatric Emergency Medicine is still an evolving discipline in Nigeria. Hospitals in Nigeria fall short of the recommendations of existing International Federation for Emergency Medicine (IFEM) and African Federation for Emergency Medicine (AFEM) guidelines/framework for running emergency departments. For instance, the IFEM recommends that the emergency departments be run by healthcare staffers that are appropriately trained and qualified to deliver emergency care; and suggests early involvement of senior doctors with specific expertise in EM for their ability to resuscitate and stabilise critical patients and to facilitate early referral to appropriate specialties. On the average, less than 50% of paediatric emergency units are headed by a dedicated Consultant Paediatrician [12, 13]. The rest are run by different paediatrician based on roster.

Despite the engagement in interactive, scenario-based courses such as the Emergency Triage Assessment and Treatment (ETAT) developed by the World Health Organisation (WHO) [26], and the Emergency Care Assessment Tool (ECAT) developed by AFEM [27], to improve paediatric emergency care, health workers in Nigeria do not have access to the training and practice that they need and desire. Paul and Edelu reported that only 55.6% of the staff (doctors and nurses) in emergency units in Nigeria had the skills for emergency triage; and < 50% of them had the skills to use either a manual or an automated external defibrillator (AED) [13]. Poor skill for emergency resuscitation among healthcare professionals is a recurring decimal that is not peculiar to Nigeria alone but has been identified in other African countries. In a recent study in South Africa, about 20% of the doctors had never performed cardiopulmonary resuscitation (CPR) in paediatric patients; and up to 35% of them did not feel confident performing CPR in children[28]. A very important contributing factor to the problem is the non-retention of trained staff in their areas of training in many public hospitals. This is often a major challenge among the nursing cadre in most facilities in Nigeria. It is difficult to retain a nurse in the emergency unit for a long period of time even after they have acquired satisfactory level of skills to improve practice.

### Availability of emergency medicines, equipment and utilities

Emergency medicines and equipment are not readily available in many emergency units around the country. Enyuma et al. reported mean medication and equipment performance scores of 50.7% and 43.9% respectively for 34 health facilities in northern and southern Nigeria[12]. The Southern region had significantly higher equipment score (47.6%) than the Northern region (38.9%) [12]. Lack basic equipment (including defibrillators) has been reported not just in Nigeria [12, 13]; but in other African countries [21, 24]. In terms of utilities, less than 50% of the facilities in Nigeria had regular running water [13]. Minimal improvement in water reticulation and supply has been noted in some hospitals.

## Challenges of paediatric emergency medicine practice in Nigeria

Healthcare providers’ experiences with paediatric emergency medicine practice in Nigeria support the need for a total reorientation and revamping of emergency medicine practice to optimize service delivery [12, 13, 29]. Despite the efforts being made by concerned stakeholders (individuals, non-governmental agencies/institutions, policy-makers, and the federal government of Nigeria) to improve emergency service delivery in the country, a lot still needs to be done to achieve the desired goal. The ultimate goal is to institute a system/framework that will deliver a sustainable high quality emergency service to Nigerian children. This can only be achieved if the numerous challenges are promptly addressed. While poor staff training, insufficient equipment, and lack of local disease-specific guidelines have been identified as the key challenges, other challenges are highlighted to direct the policy makers and stakeholders to specific areas that required urgent attention. First-hand accounts of challenges of emergency service delivery in Nigeria were well captured in published studies [12, 13, 29]. Numerous challenges were identified including poor coordination and collaboration among essential stakeholders including government and non-governmental agencies and institutions relevant to emergency care delivery[29].

The challenges include:

### Management in the community

Emergency care for the sick child starts in the community with care seeking by the parents, then to community health workers being able to recognise severe illness as the first responders [7]. The pre-hospital management of sick children at home and the subsequent transportation of the children to the desired health facility greatly impact on the outcomes of treatment for such children. Mothers especially play a key role in identifying signs of illness in their children and giving the initial home treatment. While most mothers in Nigeria irrespective of their level of education can identify a sick child, many delay presentation to health facilities and are unable to correctly institute the necessary home treatment. Abdulraheem and Parakoyi observed that mothers of sick children in a rural Nigerian setting used home remedies in up to 69.6% of reported episodes of their children’s illness. The use of health facility was consistently low (5.7–9.9%), and appropriate care was sought by only 25.3% of these mothers [30].

On the other hand, community health workers play vital roles in managing sick children at the community level. Their ability to detect signs of serious illness in children and promptly refer to the next level of health care substantially determines the health outcome. The community health workers are often trained to recognise the danger signs in line with the Integrated Management of Childhood Illness (IMCI) guidelines but they lack the appropriate skills to intervene. As such they are taught to refer to the next level of healthcare. It becomes challenging where the sick child requires an immediate life-saving intervention and the healthcare worker lacks the skills to provide such intervention.

### Referral and transport of sick children

A well-coordinated referral and transport system is key, and desirable in the Nigerian health sector. Unfortunately referral systems and between-facility transport of patients in Nigeria are still rudimentary and vulnerable. None of the states in Nigeria have functional emergency medicine services (EMS) open to the public because of the high out-of-pocket expenditure associated with the services. Emergency medicine services are virtually non-existent at primary and secondary care levels. It becomes quite challenging to transport sick children from home, health centres and district hospitals, particularly those situated in remote areas, to the nearest tertiary health facility or referral hospital for treatment. While most tertiary hospitals in Nigeria have ambulance vehicles, majority of these ambulances are ill-equipped and non-functional thus limiting the capacity to transport patients in and out of the hospitals. The situation is even worse considering the poor state of the existing road networks in Nigeria which make road ambulance service inefficient. Air ambulance service is limited to a few corporate organisations such as oil companies. Besides the limited availability of vehicle and air ambulance, training of paramedics for efficient transfer of critically ill children between facilities are largely limited and needs to be rejuvenated.

### In-hospital care

The factors accounting for delivery of sub-optimal in-hospital care to paediatric patients are multi-pronged. One of the major challenges to the timely care of the sick children is the disproportionate ratio of doctor and nurses to patients [12, 13]. The documented number of doctors (7 to 22) and nurses (10 to 24) with a nurse: bed ratio of 1:3 in children emergency units in Nigeria are grossly inadequate [13]. This ultimately results in longer waiting time and sometimes poorer attention from the healthcare providers increasing the tendency for high incidence of medical errors. Low numbers of workforce and poor distribution of qualified professionals in hospitals are general problems in less developed countries [2, 31]. These constraints make the provision of quality health care challenging in these countries. The problem is compounded by the fact that doctors and nurses in district and teaching hospitals in these countries including Nigeria have inadequate knowledge of guidelines and reported practice for managing important childhood illnesses [13, 28].

In addition to inadequate numbers of doctors and nurses in the emergency room, lack of skilled workforce has led to poor quality of services in paediatric emergency units in Nigerian hospitals.

### Policy prioritization, implementation and funding of health service

Due to poor implementation of health policies and lean healthcare funding, most paediatric emergency units in Nigeria struggle to maintain efficient services. Paediatric emergency physicians under the umbrella bodies of Society of Emergency Practitioners of Nigeria, and the Paediatric Association of Nigeria have continued to advocate for improved paediatric emergency services in the country. There is an obvious dis-connect between the various tiers of government in health system governance in Nigeria. The focus of health system challenges and solutions identified by doctors who work in the emergency room during a focused group discussion centered on the functions of the government and its responsibility in facilitating healthcare access and financing [29]. Health care financing in Nigeria is poor and undermines the desire to achieve universal health coverage for the Nigerian populace, particularly children. Presently, out-of-pocket expenditure accounts for over 70% of national spending on health [32], due to poor implementation of Nigeria’s health insurance scheme. Payment is almost 100% for all emergency services at the time the services are provided [33]. Caregivers are required to purchase every medicine and material required for resuscitation and stabilization. For example, caregivers are required to pay for blood transfusion services before these services are provided. Failure to pay for these services ultimately affects the timeline for the intervention and the quality of care. In order to avail the sick children opportunity of getting appropriate treatment within the first hour of presentation, an effective health insurance scheme at all levels of care must be instituted. Currently, Nigeria’s health insurance scheme is not functional and needs to be revamped. The identified challenges, and practical ways of surmounting them have been summarised in Table 1.

**Table 1:** Challenges of Emergency Medicine Practice in Nigeria and the way forward

Challenges of Emergency Medicine Practice in Nigeria	The way forward
Inadequate Capacity (human resources and equipment)	
• Inadequate numbers of trained emergency personnel/unavailability of accredited paediatric emergency medicine training centers	Institute formal training programmes in accredited centers to educate and train different cadres of hospital staff in skills for pediatric emergency care and life support practices to improve child survival.
Poor standard of care	
• Inappropriate management of children at home/community	Organise health education programmes for parents/caregivers of children particularly, mothers on appropriate home treatment and need for early presentation to the health facility. Institute skill acquisition programmes for community health workers with emphasis on simple life saving interventions that may be useful while taking necessary steps to facilitate referral at appropriate time.
• Lack of coordinated referral and transport of sick children	Institute a well-coordinated referral, communication, and transport system between facilities to enhance early and safe arrival of the sick child to the facility for appropriate intervention.
• Inadequate numbers/distribution of health personnel in the hospitals	Employ adequate numbers of health workers to cater for the patients in the emergency units.
• Unavailability of basic equipment	Provide basic equipment needed to improve quality of care and outcome of childhood illnesses.
Poor policy prioritization/implementation and funding of health care services	
• Lack of prioritisation and implementation a country-wide policy for paediatric emergency medicine practice	Institute a well-planned policy on paediatric emergency service and hold relevant ministries/parastatals and regional health authorities accountable for proper implementation.
• Poor funding of health care and dysfunctional health insurance scheme	Increase health care budget and institute a functional health insurance scheme in Nigeria to ensure free health care services to the disadvantaged population of children.
• Enormous out of pocket expenses	Harmonize expenditure/billing system such that the life of the child is prioritised over immediate need for out of payments by caregivers.
• Skewed orientation/provision of health service in favour of adults	Ensure equity in service by fostering more child oriented health services.

## Improving paediatric emergency medicine practice in Nigeria: the way forward

### Policy prioritization and funding of health service

There is often a culture of ignorance or acceptance of poorer standards of care by health workers with the ardent hope that policy makers will see the urgent need to totally revamp the health systems in Nigeria in the future. Indeed, improvement in paediatric emergency service in Nigeria strongly depends on effective policy development, prioritization, and implementation. A system-wide paediatric emergency care planning, preparedness, coordination, and funding are key to enthronement of minimum standards of care in paediatric emergency care. The pre-hospital system needs improvement. An emergency management system must be carefully planned with the involvement of the relevant national ministries and sub-national health authorities [7]. The health care service and referral system among the primary, secondary and tertiary levels of care in Nigeria are often uncoordinated [34]. Thus, services in many tertiary hospitals have usurped those of the primary and secondary health facilities. Services within the hospitals are poorly coordinated such that paediatric departments struggle for survival where the services are to a greater extent driven by adult oriented policies and regulations. The situation can only be improved with the development and implementation of goal-oriented policies and strategic frameworks that equally cut across both adults and children. Relevant organisations such as the Paediatric Association of Nigeria who are advocates of child health care in Nigeria should influence policy formulation and implementation in paediatric emergency services. Improved health care financing is desirable.

### Capacity development

Training of health workers in all health care facilities including those in the rural communities is an integral part of the child survival strategies globally. Regular pediatric life support training for emergency practitioners both at primary, secondary and tertiary care facilities will enhance child survival at every encounter. There is a need to bolster paediatric emergency medicine practice through education and training of different cadres of hospital staff in pediatric emergency care to ensure more optimal outcome [35]. Hence, establishing paediatric emergency medicine training programmes for physicians, nurses, and pre-hospital personnel becomes imperative.

Introducing a well-designed paediatric emergency medicine skill-based learning programme into the various medical curricula in Nigerian Universities on a broader scope may be the best approach to lay a solid foundation for improved emergency service delivery in Nigeria. This will go a long way to sharpen the students’ skills and preparedness for emergency medicine practice in the future.

In 2011, the IFEM developed model curriculum for emergency medicine specialists training. This document defined the basic minimum standards for specialist trainees in emergency medicine [36]. Subsequently, the Paediatric Emergency Medicine Special Interest Group (PEMSIG) of the IFEM produced a document applicable on a global level, which delineates valuable practical standards for care of children in emergency settings. [37]. This document recognizes the varying challenges inherent in different parts of the world, including differences in patient load, burden of disease, staffing, infrastructure, and access to education in pediatric emergency care, equipment, and medications. Similarly, the African Federation of Emergency Medicine (AFEM) developed a curriculum tailored for paediatric emergency medicine training in Africa [38]. Using these resourceful documents, relevant stakeholders involved in paediatric emergency care in Nigeria should as a matter of urgency pursue the agenda to begin a subspecialist training programme in Nigeria to bring about the long awaited change in paediatric emergency practice in the country. The quality of training could be checked by relevant bodies such as the postgraduate medical colleges of Nigeria to maintain the required standards for such training. While plans are already initiated to start such training programmes, there is need to encourage all health care workers to seek for opportunities for training where they exist.

### Improving standard of care

The quality of service can be enhanced by ensuring that minimum standards for emergency care are maintained in all hospitals in Nigeria. The PEMSIG consensus document assists hospitals around the world in defining minimum standards of care for children aged 0–18 years in the Emergency Department [37]. This comprehensive document could serve as a guide to improve services in Nigeria within the local context. There is need to develop standard treatment protocols and provide basic equipment that will enhance quality of service.

In order to help emergency practitioners in Africa to identify gaps in quality of care delivery in their various hospitals a group of experts who work in the African settings derived a simple, practice-based quality assessment tool (PBT) for resource-limited settings aimed at improving the management of sentinel emergency presentations in children [1]. The PBT is essentially a list of actions including core skills in initial assessment and management of an ill child in emergency setting within the first hour of care. The absence of these actions in the hospitals reflects a modifiable gap in the quality of care delivery. Like the PEMSIG and AFEM guidelines for maintaining quality of care in paediatric emergency settings, the PBT tool may be used to assess the availability of minimum expectation for care in those centers where resources are very limited. The PBT also helps to identify both individual and collective need for training and re-training; and measure the impact of a change in practice following an education or policy intervention within a department. A useful approach to improving paediatric medical systems identified by Khan and colleague include the establishment of a coordinated approach to patient care, and increased inter-departmental cooperation and collaboration within hospitals [39]. In a hospital in Lilongwe Malawi, Simple, inexpensive interventions such as posting of senior doctors to supervise pediatric services in under-five clinics, institution of a formal triage process that improved patient flow, and treatment and stabilization of patients before transfer to the inpatient ward improved pediatric emergency care and decreased hospital mortality rates [11]. Such simple but effective interventions can be enthroned in paediatric departments in Nigeria. Regular trainings of staffs in skills such as Paediatric Basic and Advanced life Support are imperative in order to bridge the existing training gaps and improve the overall practice.

In conclusion, paediatric emergency medicine is a critical aspect of paediatrics that impacts child survival, morbidity and mortality rates in Nigeria, and other LMIC countries. To this end, concerted efforts by all the stake holders including paediatricians, government and non- governmental organizations, and hospital administrators are needed to drive the progress of paediatric emergency practice in Nigeria. Important aspects of intervention to improve services include capacity development, improved funding of paediatric practice, and provision of basic equipment for emergency care. There should be equitable redeployment of the available resources to the major areas of need in the hospitals to optimize service delivery.

## Data Availability

Not applicable.

## Abbreviations

AED: Automated External Defibrillator
AFEM: African Federation of Emergency Medicine
APFP: African Pediatric Fellowship Program
ECAT: Emergency Care Assessment Tool
ED: Emergency Department
ETAT: Emergency Triage Assessment and Treatment
IFEM: International Federation for Emergency Medicine
LMICs: Low-Middle Income Countries
PBT: Practice-based Quality Assessment Tool
PEDs: Paediatric Emergency Departments
PEM: Paediatric Emergency Medicine
PEMSIG: Paediatric Emergency Medicine Special Interest Group
PICU: Paediatric Intensive Care Unit
SSA: Sub-Saharan Africa
WHO: World Health Organization
